# Vaginal Fluid Urea and Creatinine in the Diagnosis of Premature Rupture of Membranes in Resource Limited Community Settings

**Published:** 2017-03

**Authors:** Jasmina Begum, Sunil Kumar Samal, Seetesh Ghose, Gopal Niranjan

**Affiliations:** 1 Great Eastern Medical School and Hospital, Ragolu, Srikakulum, Andhra Pradesh, India; 2 Department of Obstetrics and Gynecology, Mahatma Gandhi Medical College and Research Institute, Pillaiyarkuppam, Puducherry, India; 3 Department of Biochemistry, Mahatma Gandhi Medical College and Research Institute, Pillaiyarkuppam, Puducherry, India

**Keywords:** Premature Rupture of Membranes (PROM), Urea, Creatinine, Vaginal Discharge

## Abstract

**Objective:** Diagnosis of premature rupture of membranes (PROM) is difficult in equivocal cases with traditional methods. This study aimed to evaluate the reliability of vaginal washing fluid urea and creatinine for diagnosis of PROM and to determine the cut off value.

**Materials and methods:** The current study was a prospective case control. Women having gestational age of 28 to 42 weeks were divided into two equal groups: Fifty with history of leaking per vagina (study group) and an equal number with gestation matched none leaking (control group) were recruited. Data analysis was done by Student’s t-test, receiver operator curve and chi square test.

**Results:** The demographic data of both groups were comparable at the time of sampling (p > 0.05).Vaginal fluid urea and creatinine was significantly higher in study group (p < 0.001). The sensitivity, specificity, positive predictive value (PPV), negative predictive value (NPV), and accuracy of vaginal fluid urea with a cut off value > 6mg/dl and creatinine with a cut off value of > 0.3 mg/dl to diagnose PROM were all more than 90%. The sensitivity, specificity, PPV, NPV and accuracy of amniotic fluid index (AFI) to diagnose PROM were 30%, 91.8%, 83.33%, 57.32% and 62 % respectively, with a cut-off value of ≤ 7 cm. The areas under the curves are 0.952 for creatinine, 0.999 for urea and 0.635 for AFI.

**Conclusion:** Detection of vaginal fluid urea and creatinine to diagnose PROM is a simple, reliable and rapid test. Introduction of this method into routine use even in low resource community setting is feasible, practical and cost effective.

## Introduction

Premature rupture of membranes (PROM) refers to rupture of the fetal membranes prior to the onset of labor, regardless of gestational age. It occurs in 10% of all term pregnancies and about 2- 4% of preterm pregnancies, and is associated with complications such as infection and preterm birth (1). Accurate history, clinical examination and specialized tests are the hallmark for diagnosing PROM. Failure to identify patient or false positive diagnosis of PROM may lead to inappropriate management and serious maternal and neonatal complications or unnecessary obstetric interventions (2). 

Diagnosis of PROM can be made by showing the three gold standards of conventional findings by a clinician (3); 1) Observation of clear amniotic fluid flow or accumulation of fluid at posterior fornix with a sterile speculum, 2) Observation of transition from yellow to blue with pH indicator paper due to basic amniotic fluid flow (nitrazine test) and/or 3) Detection of palm leaf-pattern in dried amniotic fluid with microscopic method (fern test). 

However these conventional methods are associated with drawbacks. History is reliable in 10% to 50% of cases speculum examination of fluid leakage from the cervix was associated with 12% - 30% false negative results, Nitrazine test was associated with false positive or negative results due to contamination by urine (alkaline), blood or meconium, antibiotics, vaginal and cervical infections, and fern test was also associated with 13-30% false negative and 5-30% false positive results (4). Even amniotic fluid determination by ultrasound examination was not a reliable test to evaluate membrane rupture because it cannot differentiate PROM from other causes of oligohydramnios (5).

Intra amniotic dye injection and observation for fluid passage transvaginally was designated an “unequivocal” diagnostic method for confirmation of membrane rupture, but this invasive test carries increased maternal and fetal risk (6). The Amnisure ROM test is another new test that is easy, fast, and minimally invasive, with high sensitivity and specificity (7, 8). However, Amnisure is not available in many centers and it is expensive.

Many biochemical diagnostic modalities for PROM have been described, like measurement of vaginal PH, alpha fetoprotein (AFP), insulin growth factor binding protein-1(IGFBP-1), fetal fibronectin tests, human chorionic gonadotropin (HCG), prolactin, urea, creatinine (4,5, 9-13).

The rationale of assessing these markers stems from their high concentrations in amniotic fluid compared with normal vaginal secretions. These tests are based on the identification in the vaginal washing of one or more of these biochemical markers that are present in the setting of PROM, but absent in women with intact membranes. However, despite the improved diagnostic potential of these markers, they have not become popular due to their complexity and high cost (9).

Recently, the focus has been on urea and creatinine in cervicovaginal discharge. Measurement of urea and creatinine in the vaginal fluid for the diagnosis of PROM is based on the concept that fetal urine is the prime component of amniotic fluid in the second half of pregnancy. The fetus starts excreting urine into the amniotic fluid at 8^th^ to 11^th^ week of gestation. As there is no need for extra equipment and reagent, introduction of this method into routine use is feasible and practical. So, the present study is designed to evaluate the reliability of vaginal washing fluid urea and creatinine for diagnosis of PROM and to determine the cut off value for urea and creatinine in vaginal washing fluid.

## Materials and methods

It is a Prospective study on diagnostic test for PROM, performed in the Department of Obstetrics and Gynaecology of Mahatma Gandhi Medical College and Research Institute, Puducherry, India. This study got the approval of the local ethics committee and an informed consent was taken from all pregnant women who participated in the study. 

The sample size was determined based on accuracy level of diagnosis of PROM by the measurement of vaginal fluid urea and creatinine, which was found to be 85% in the previous studies. Hence, from the available information the sample size is calculated to 100 with 80% of power at 95% confidence level.

Women with singleton live pregnancy between 20-34 years of age, at gestational age of 28-42 weeks (by LMP or first trimester sonography finding) were studied. Pregnant women with vaginal spotting/ bleeding, meconium in the vaginal fluid leak, recent vaginal infection having history of use of vaginal drugs, pregnant women with presence of regular uterine contractions, pregnant women with known medical and prenatal complications, pregnant women with cohabitation in the prior night, and with fetal anomalies were excluded. 

All the women who participated in study were subjected to full history taking, general and abdominal examination and sterile speculum examination to confirm amniotic fluid flowing from the cervix. A cotton tip applicator inserted in deep vagina was transferred on nitrazine paper. If the nitrazine paper turned from yellow to blue colour, it was considered as positive for amniotic fluid leak from the cervix.

Fifty pregnant women, who have complains of vaginal fluid leakage, and in whom PROMwas diagnosed via positive fluid leak upon sterile speculum examination and positive nitrazine paper test, were taken through non-probability (convenience) sampling as study group (Group 1).

Meanwhile, among pregnant women admitted to antenatal ward for their regular prenatal control visit, 50 pregnant women with matched gestational age, with negative fluid leak upon sterile speculum examination, and with negative nitrazine paper test were taken as control group (Group 2).

All patients underwent transabdominal sonography for GA, AFI, and fetal viability. Thereafter, vaginal washing fluid urea and creatinine sampling was done as follows. 5 ml of sterile saline solution will be injected into the posterior vaginal fornix and 3 ml of it will be withdrawn with the same syringe. 

One of our authors carried out sterile speculum examination for diagnosis of PROM. Sampling procedure was performed within 6 hour after membrane rupture and before vaginal examination or administration of any drugs. Samples were sent to the laboratory for measurement. Demographic and obstetric characteristics, results of speculum examination, nitrazine test, urea and creatinine, as well asultrasonographic finding of AFI were documented in a data form. Urea level was measured by enzymatic urease method and a creatinine level was measured by Jaffee chemical calorimetric method, respectively. 

***Statistical analysis:*** Description of quantitative (numerical) variables was performed in the form of mean, standard deviation (SD) and range. Description of qualitative (categorical) data was performed in the form of number and percent. Analysis of numerical variables was performed by using independent student’s t-test and categorical data by using Chi-squared test. Diagnostic accuracy was assessed using the following terms: sensitivity, specificity, positive predictive value (PPV), negative predictive value (NPV) and overall accuracy. 

Receiver operating characteristic (ROC) curve analysis was used to establish an optimal cut‐of concentration. The results were evaluated with a significance level of p < 0.05.

## Results

A total of 100 pregnant women were included in the study. They were divided into 2 groups according to presence or absence of PROM, Group I (cases): included fifty pregnant women with PROM. Group II (controls): included fifty pregnant women without PROM. Demographic data for study groups is represented in table 1. There was no statistical significant difference between both groups regarding, maternal age, gravid, parity and gestational age at time of sampling, both the groups were comparable (p > 0.05) (Table 1).

There was a statistical significant difference between the 2 groups regarding vaginal fluid urea. In the current study, the mean value of urea was higher in the study group than in the control group (12.60 ± 4.85 vs. 2.12 ± 1.31, p < 0.001). The sensitivity & the specificity of vaginal fluid urea to diagnose PROM were 98% & 100% respectively. While itsPPV, NPV, over all accuracy were 100%, 98.4%, 99% respectively, with a cut-off value > 6 mg/dl (Tables 2, 3).

There was a statistical significant difference between the 2 groups regarding vaginal fluid creatinine. The mean value of creatinine was higher in the study group than in the control group (0.67 ± 0.31 vs. 0.16 ± 0.09, p < 0.001). The sensitivity & the specificity of vaginal fluid creatinine to diagnose PROM were 90% & 93.83% respectively, while its positive predictive value (PPV), negative predictive value(NPV), and over all accuracy, were 97.83%, 90.74% and 94 % respectively, with a cut-off value of >0.3 mg/dl (Tables 2, 3).

**Table 1:** The demographic characteristics of groups

**Parameters**	**PROM (+)**	**PROM (-)**	**p Value**
	**Group I (n = 50)**	**Group 2 ( n = 50)**	
Maternal Age (Years)	23.90 ± 3.67	22.92 ± 4.25 (NS)	0.220
Gestational Age at sampling (weeks)	37.70 ± 1.90	37.42 ± 4.46 (NS)	0.684
Gravida	1.48 ± 0.67	1.52 ± 0.73	0.775 (NS)
Parity	0.42 ± 0.60	0.42 ± 0.70	1.00 (NS)

**Table 2:** Vaginal fluid urea and creatinine level (mg/dl) and AFI (cm) among groups

**Parameters**	**PROM (+)** **Group I (n = 50)**	**PROM (-)** **Group 2 (n = 50)**	**p Value**
Vaginal fluid urea ( mg/dl)	12.60 ± 4.85	2.12 ± 1.31	< 0.001**
Vaginal fluid Creatinine ( mg/dl)	0.67 ± 0.31	0.16 ± 0.09	< 0.001**
AFI	8.41 ± 2.91	9.56 ± 2.61	0.041*

** Strongly Significant,

* Moderately significant

AFI difference was just statistically significant between the two groups. The mean value of AFI was lower in the study group than in the control group (8.41 ± 2.91 vs. 9.56 ± 2.61, p = 0.04), The sensitivity & the specificity of amniotic fluid index (AFI) to diagnose PROM were 30% & 91.84% respectively, while it’s PPV, NPV, and over all accuracy, were 83.33%, 57.32% and 62 % respectively, with a cut-off value of ≤ 7 cm (Tables 2, 3).

**Figure 1:**
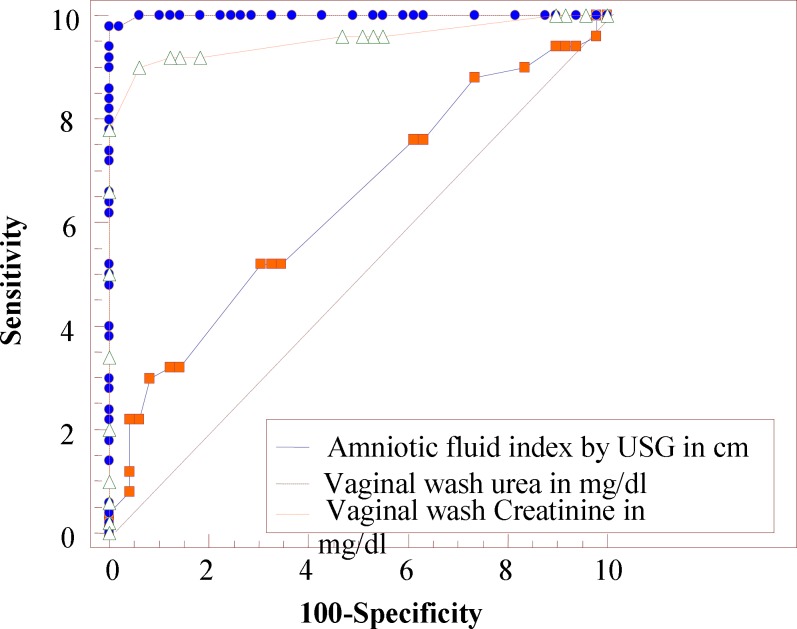
Receiving operator characteristic curve for vaginal wash urea, creatinine levels and AFI

Receiver operating characteristic (ROC) curve analysis was used to establish the optimal cut-off concentrations for vaginal washing fluid urea, creatinine and AFI. The areas under the curves are 0.952 for creatinine, 0.999 for urea and 0.635 for AFI (Table 3, Figure 1).

## Discussion

The importance of proper diagnosis of PROM cannot be further stressed as it can act as double edge sword. Inability to diagnose in time can lead to obstetric complications like cord prolapse, abruption, chorioamniotis, postpartum endometritis, neonatal sepsis and on the other hand over diagnosis can lead to unnecessary hospitalization, induction of labor and preterm birth. Amniotic fluid, maternal blood and urine contain urea and creatinine. Pregnant women in the first half of pregnancy have a mean creatinine concentration of 0.6mg/dl in the amniotic fluid which is similar to maternal serum (14, 15).Gradual increase of creatinine concentration is noticed in amniotic fluid between 20 and 32 weeks of gestation, which rises even more rapidly to reach a value as high as two to four times that of maternal serum (14, 15).

A creatinine concentration of 1.75mg/dl or more correlates significantly with a gestational age of 37 weeks or more (15).Therefore, it can be surmised that vaginal urea and creatinine can be used not only in the diagnosis of PROM but also for fetal maturation test in case of preterm labor. Reliability of conventional test does not match with the patient’s leaking history, so the search for simple, fast, easily accessible and non-invasive diagnostic methods to detect rupture of membranes is still in vogue. This study considers testing the diagnostic accuracy of urea and creatinine in vaginal fluid, which can be easily accessible in low resource community settings for PROM.

**Table 3:** ROC curve analysis

**Parameters**	**ROC Results**				**Cut off**	**AUROC**	**p value**
	**Sensitivity**	**Specificity**	**PPV**	**NPV**			
AFI(cm)	30.00	91.84	83.33	57.32	0.014*	≤7.0	0.635
Vaginal							
Fluid Urea (mg/dl)	98.00	100.00	100.00	98.4	<0.001**	> 6	0.999
Vaginal fluid							
Creatinine (mg/dl)	90.00	93.83	97.83	90.74	<0.001**	> 0.3	0.952

** Strongly Significant,

* Moderately significant

Our study showed that both vaginal fluid urea and creatinine concentrations are good predictors of PROM. The best cutoff point for vaginal fluid urea concentration as diagnostic of PROM was > 6 mg/dl (sensitivity 98% specificity 100%). The best cutoff point for vaginal fluid creatinine concentration as diagnostic of PROM was > 0.3 mg/dl (sensitivity 90% specificity 93.8 %). 

Oligohydramnios may not be detected in patients with confirmed PROM, possibly because drainage may become intermittent or even stop once the presenting part descends and acts as a plug, preventing further drainage. The cutoff point for AFI as diagnostic of PROM was ≤ 7cm (sensitivity 30 % specificity 91.8 % accuracy 62%). Present study has shown that a reduction in the four-quadrant AFI ≤ 7cm is not reliable in identifying cases of membrane rupture by history, with visualization of fluid in speculum examination and positive nitrazine paper. This shows that ultrasound quantification of AFI has poor quality in diagnosing membrane rupture. Receiver Operator Curve (ROC) analysis indicates that urea has higher specificity, sensitivity, positive and negative predictive values, and accuracy than other two markers, creatinine and AFI for PROM in the current study. 

Kafali et al. were first to use urea for diagnosis of PROM. In their study, 47 patients with confirmed PROM, 36 patients with suspected but unconfirmed PROM and 50 pregnant women without any complaint or complication were included. The sensitivity, specificity, NPV and PPV were all 100% in detecting PROM by evaluation of vaginal fluid urea and creatinine concentration with cut off values of 12 and 0.6mg/dl respectively (15). Mohamed et al. had also found 100% sensitivity, specificity, NPV and PPV of evaluation with cut off of urea 13.2 mg/dl and creatinine 0.31mg/dl in detecting PROM (16).

Zanjani et al. studied the reliability of vaginal fluid creatinine for the diagnosis of premature rupture of membranes. They compared in 3 groups, 60 confirmed PROM patients, 60 suspected and 60 controls. The creatinine level was significantly higher in the confirmed group than in the other two groups (P < 0.0010). They found 96.7% sensitivity, 100% specificity, 100% PPV, 96.8% NPV with optimal cutoff value of 0.5mg/dl. They concluded that it is a valid and simple test, especially considering that it costs much less than hospitalization or other tests (2).

Kariman et al. also studied 2 groups to compare vaginal fluid urea and creatinine in confirmed cases and normal controls. They found the mean level of vaginal fluid urea and creatinine in the PROM group to be significantly higher than in the control group. A similar observation was seen in the present study where a high level of urea and creatinine was found among the study group which was statistically significant (P < 0.001) (4).

Kariman et al. subsequently studied 3 groups, 60 confirmed PROM patients, 66 suspected and 53 controls. In their study they demonstrated that measurement of vaginal fluid urea and creatinine is a simple and reliable test for diagnosis of PROM. For creatinine with a 0.45 mg/dl cut-off point had higher sensitivity and specificity than urea with a 6.0 mg/dl cutoff point and they concluded that the vaginal fluid creatinine to be the gold standard for diagnosing PROM. However, in our study creatinine levels had less diagnostic value than urea levels contrary to the above study which can be attributed to the different cut-off points for creatinine or smaller sample size (17).

Osman et al. studied 3 groups, 50 confirmed PROM patients, 50 suspected and 50 controls and compared AFI and vaginal fluid urea, creatinine and β-hCG. They showed a statistically significant difference among all groups regarding AFI, urea, creatinine and β-hCG levels. They found sensitivity, specificity, PPV, NPV and accuracy of urea and creatinine were all 100% with a cut off value of > 0.41 mg/dl and > 0.23 mg/dl. They concluded urea and creatinine in vaginal washings can be accurately used in diagnosing suspected PROM and are more accurate than β-hCG (18).

Tigga et al. studied 2 groups, 50 confirmed PROM patients and 50 controls, and compared vaginal fluid alpha fetoprotein, prolactin creatinine and β-hCG. They found that all the four markers were significantly higher in the patients with PROM in comparison to those without PROM. They found sensitivity, specificity, PPV, NPV and efficiency of creatinine were 100%, 92%, 92.59% , 100% and 96% efficiency with a cut off value of 0.1641 mg/dl and Creatinine was reported to be simple, cheap, reliable and easily available method for diagnosing PROM (12).

Ghasemi et al. studied in 2 groups, 80 confirmed PROM patients and 80 controls and compared vaginal fluid prolactin, β-hCG, urea, and creatinine. They also showed that all the four markers were significantly higher in the patients with PROM in comparison to those without PROM (p < 0.001) .An optimal cutoff level of 3.5 mg/dl for urea and 0.25 mg/dl for creatinine was seen in their study. The sensitivity, specificity, positive and negative predictive values to diagnose PROM were calculated and found to be as 79.7%, 82.5%, 81.8% and 80.4% for urea and 74.6%, 85%, 83% and 77.2% for creatinine. They reported less diagnostic value of urea and creatinine for detecting PROM, because of the difference in laboratory method analysis and cut off points (13).

Gezer et al. conducted a study on 200 patients of which half were controls and other half were confirmed PPROM cases. An optimal cutoff level of 6.7 mg/dl for urea and 0.12 mg/dl for creatinine was seen. The sensitivity, specificity, positive and negative predictive values to diagnose PPROM were calculated and found to be as 88%, 91%, 90.7% and 88.3% for urea and 89%, 90%, 89.9% and 89.1% for creatinine, respectively. Analyzing the ROC curve showed an area under the curve for urea as 0.944 and for creatinine as 0.902. They finally came up with a suggestion that urea levels > 6.7 mg/dl and creatinine levels> 0.12 mg/dl in vaginal fluid, can diagnose PPROM in approximately 88% and 89% of women respectively, if seen between 24 and 37 weeks. They also hinted that in patients with shorter delivery interval, raised vaginal urea and creatinine levels may suggest a large defect in amniotic membrane or increased production by fetus(19).

The strengths of our study were that we used more than one criterion to diagnose PROM. They included symptoms, signs and investigations. Factors interfering the test were controlled by following strict inclusion criterion, prospective nature of study design, and sampling procedure done by one author. Limitation of the study was lack of comparison between vaginal fluid urea, creatinine and the other diagnostic tests that are available as the present study was none funded.

## Conclusion

Detection of vaginal fluid urea and creatinine to diagnose PROM is a simple, reliable and rapid test with high sensitivity, specificity, PPV, NPV and over all accuracy. Introduction of this method into routine use is feasible and practical as there is no need for extra equipment and reagents. It is available even in the low resource community settings and is cost effective for PROM. It can be used particularly as an adjunctive test in equivocal cases of PROM. The difference in the cut-off levels between the various studies may be attributed to the different sample sizes, inclusion criteria and the gestational age of studied patients. We suggest further studies can be taken up with different gestational age groups for determination of cut-off values of vaginal wash urea and creatinine for diagnosing rupture of membranes in pregnancy.
